# Analysis of endogenous lipids during intestinal wound healing

**DOI:** 10.1371/journal.pone.0183028

**Published:** 2017-08-11

**Authors:** Yunna Lee, Jieun Choo, Su Jin Kim, Gwangbeom Heo, Charalabos Pothoulakis, Yong-Hak Kim, Eunok Im

**Affiliations:** 1 College of Pharmacy, Pusan National University, Busan, Republic of Korea; 2 Section of Inflammatory Bowel Disease & Inflammatory Bowel Disease Center, Division of Digestive Diseases, David Geffen School of Medicine, University of California Los Angeles, Los Angeles, California, United States of America; 3 Department of Microbiology, Catholic University of Daegu School of Medicine, Daegu, Republic of Korea; Lewis Katz School of Medicine at Temple University, UNITED STATES

## Abstract

Intestinal wound healing is a new therapeutic goal for inflammatory bowel disease (IBD) as complete healing of the mucosa is the key element of clinical remission in IBD. Previous studies showed that termination of inflammation can be achieved by adding pro-resolving lipids like DHA and EPA exogenously. However, the roles of these lipids in mucosal healing have not been investigated. To recapitulate intestinal healing process, mice were received dextran sodium sulfate (DSS) for 7 days in the drinking water followed by regular tap water for 5 additional days. DSS-induced intestinal inflammation featuring body weight loss, histological tissue damage, increased cytokine production and infiltration of inflammatory cells was gradually reduced upon switching to water. To investigate whether endogenous lipids play a role in mucosal healing, the lipidomics analysis of mouse serum was performed. Reduced levels of arachidonic acid, the biosynthetic precursor of prostaglandin F (PGF)_2α_, 19H-PGF_1α_, the metabolite of prostacyclin, and 20H-PGF_2α_, the metabolite of PGF_2α_, suggest subsiding inflammation. In contrast, increased levels of an active metabolite of resolvin D1 along with decreased levels of its precursor DHA as well as decreased levels of the precursor of resolvin E, 18-hydroxy-eicosapentaenoic acid, suggest inauguration of mucosal healing by endogenous lipids. Furthermore, exogenously supplied fish oil enhanced the process even further. These results suggest the presence of mucosal healing regulated by endogenous pro-healing lipids and also indicate that the remission state of IBD could be prolonged by enhancing the levels of these lipids.

## Introduction

Inflammatory bowel disease (IBD) that includes Crohn’s disease and ulcerative colitis is chronic debilitating conditions of unknown etiology. The incidence of IBD has considerably increased in the last century, now representing a common chronic inflammatory disease worldwide [1]. Typically, IBD is marked by repeated relapses and remissions over long periods of time. According to the newest reports, at least 30% of IBD patients experience more than one episode of relapse per year [2, 3]. Therefore, maintaining stable remission without clinical symptoms and decreasing the incidence and duration of relapse are the ultimate goals of IBD treatment [4, 5]. Among the treatment approaches for IBD, anti-TNFα antibodies such as infliximab, adalimumab and golimumab are widely used and one of the most successful therapies, and induce and maintain clinical responses in patients with IBD [6–8]. Despite of important clinical efficacy of these agents, anti-TNFα antibodies may limit the host immune system and result in undesirable side effects including lymphoproliferative disorders and opportunistic infections [9, 10]. Recent studies have also reported that some patients become non-responsive and/or show a poor response to anti-TNFα therapies [11–13]. These necessitate the development of new therapeutic strategies.

Inflammation is primarily a host defense response to protect against pathogens and tissue injury. Polymorphonuclear leukocytes (PMNs) are the first effectors recruited to the inflamed sites and have a critical role in immune defense [14]. Despite the beneficial role of PMNs to the host, their inappropriate activation leads to tissue damage and exaggerated inflammatory reactions. Therefore, inflammation is gradually terminated by inhibiting activation of PMNs, clearing infections, and repairing tissue injury. Termination of inflammation has a major role in maintaining homeostasis of the host immune system through an actively regulated cellular program known as resolution [15, 16]. If the resolution process is dysregulated, inflammation persists and contributes to the pathology of many chronic inflammatory diseases and autoimmunity such as rheumatoid arthritis, atherosclerosis and IBD [17, 18].

Therefore, many recent studies have focused on termination of inflammation and its regulators, specialized pro-resolving lipid mediators (SPMs), to find new therapeutic clues by using endogenous mechanisms of self-limited resolution processes [19–21]. The first SPM investigated was lipoxin, derived from arachidonic acid (ARA), an n-6 polyunsaturated fatty acids (PUFA), generated by cell to cell and lipoxygenase interactions [22]. Furthermore, mounting evidence supports that n-3 PUFA and their active lipid metabolites reduce the production of inflammatory derivatives and promote the resolution of inflammation. During active resolution, n-3 PUFA such as eicosapentaenoic acid (EPA) and docosahexaenoic acid (DHA) are used to generate SPMs including resolvins, protectins and maresins [23–25].

Because resolution of inflammation is recognized as one of the most important processes to control inflammation and maintain tissue homeostasis, many studies have focused on finding new pro-resolving and anti-inflammatory factors. However, current studies are limited to test pharmaceutical efficacy of candidate molecules and/or reagents by using only exogenous application in inflammatory diseases. Therefore, there is no information and understanding of the endogenous mediators of the resolution process in each inflammatory disease except one study showing the molecular circuits and major components of the endogenous resolution process in a mouse peritonitis model [19, 26]. To achieve successful treatment and improve patient outcomes of IBD, maintaining stable remission is critical. This could be achieved by understanding the resolution process which may induce stable remission and boosting the production of pro-resolving mediators.

Healing of the inflamed mucosa is a key step to achieve clinical remission in IBD. The structural basis of mucosal healing includes various molecular and cellular signaling pathways [27]. The healing process is initiated by migration of intestinal epithelial cells residing near the wounded area to the injury sites. Cytokine-mediated tumor growth factor-β production and chemokine-mediated changes in the actin cytoskeleton induced by the Rho family regulates cell migration and therefore promotes wound closure [28, 29]. Proliferation of epithelial cells is a next key step in wound healing and is mediated by growth factors, hormones, and cytokines. These factors mediate activations of transcription factors including nuclear factor-κB (NF-κB), STAT3 and STAT5 and eventually boost epithelial cell proliferation [30–33]. Ultimately, closure of erosions and ulcerations takes place as a final step of wound healing. Antimicrobial peptides and intestinal mucins can support this process [34, 35].

New therapeutic strategies for achieving mucosal healing have been proposed and various candidate drugs have been tested in clinical trials. Those include anti-TNF agents, antibodies against interleukins (IL-6R, IL-13, and IL12/IL-23), regulatory T cell therapy, and blockade of adhesion molecules. Therefore, many new targets to induce mucosal healing and eventually complete remission of IBD will play a key role in future therapy of IBD.

The purpose of the present study was to investigate whether resolution takes place in acute colitis and if so, to identify molecules that regulate this process. To this end, we established a wound healing model which recapitulates the remission state of IBD and investigated the main features of endogenous resolution machinery as well as endogenous pro-resolving mediators.

## Materials and methods

### Animal models

Eight-week-old, male C57BL/6 mice were purchased from Samtako Co. (Kyungki-do, Korea) and Japan Shizuoka Laboratory Center, Inc. (Shizuoka, Japan). Animals were housed in an air-conditioned atmosphere under a 12 h light: dark cycle with free access to laboratory chow and drinking water. All animal study protocols used in this study were reviewed and approved by the Pusan National University-Institutional Animal Care and Use Committee (PNU-IACUC, Busan, Korea) for ethical procedures and scientific care (Approval Number PNU-2014-0670). The methods undertaken to minimize potential pain and distress include providing enrichment such as nestlets and changing dirty cages more frequently than once per week. The clinical criteria for euthanasia include weight loss of 20–25%, decreased appetite, weakness/inability to obtain food or water, a lack of sustained purposeful response to gentle stimuli, or other signs of chronic disease.

The wound healing model after acute colitis was established as previously described [36]. The mice were randomly assigned to 7 groups (D7+W0, D7+W1, D7+W2, D7+W3, D7+W4, D7+W5 and No DSS). The mice in D7+W0~W5 groups were received 2.5% (wt/vol) dextran sulfate sodium (DSS, MW 36,000–50,000) (MP Biomedicals, Santa Ana, CA, USA) in the drinking water for 7 days followed by regular tap water for 5 additional days. No DSS group was received only tap water as a control group. For the fish oil supplementation experiment, mice were fed 100 μl of Menhaden fish oil (Sigma-Aldrich, St Louis, MO, USA) diluted in sunflower oil by oral gavage during the 5-day tap water treatment period (D10+W0~W5). The concentration of EPA and DHA in Menhaden fish oil was 0.25 g/mL, and each mouse was fed 100 μg/g of EPA + DHA daily. The control group received the same volume of sunflower oil (as vehicle) per body weight. For the DHA and EPA supplementation experiment, mice were fed 50 μg/g of EPA (Tocris Bioscience, Bristol, UK), 50 μg/g DHA (Tocris Bioscience) or 25 μg/g EPA+ 25 μg/g DHA diluted in sunflower oil by oral gavage during the tap water treatment period.

### Clinical assessment of colitis and histological evaluation

Mice were monitored daily for disease activity as previously described [37]. Briefly, clinical parameters of colitis including body weight, rectal bleeding, and diarrhea were determined daily by trained individuals blinded to the treatment information. The degree of symptoms was graded on a scale of 0–7 (Diarrhea: 0 = no diarrhea, 2 = pasty and semiformed stools, 4 = liquid stools; Bleeding: 0 = no bleeding, 2 = occult bleeding, 4 = bloody fluid with diarrhea). For histological evaluation, the colon was excised and the length was measured. In addition, the segments of the transverse colon (1 cm) were fixed immediately in 10% buffered neutral formalin solution (Sigma-Aldrich), embedded in paraffin, and stained with hematoxylin and eosin (H&E). Sections were examined with Olympus BH-2 Microscopes (Olympus Corporation, Tokyo, Japan) and photographed with Moticam 3.0 MP Color Digital Camera (Motic, Causeway Bay, Hong Kong) using Motic Images Plus 2.0 software. The histological severity of colitis was graded in a “blinded” fashion on a scale of 0–4 as previously described [37, 38].

### Immunohistochemistry

Formalin-fixed colon tissues were embedded in paraffin and sectioned at 4 to 5 μm. Slides were deparaffinized with xylene (Duksan Pure Chemicals, Kyungki-do, Korea), rehydrated with sequential washes of decreasing concentrations of ethanol (Merck Millipore Corporation, Billerica, MA, USA), and rinsed in tap water (100% xylene 5 min: 2 times, xylene 1:1 with 100% ethanol 5 min: 2 times, 95% ethanol 5 min, 70% ethanol 5 min, 50% ethanol 5 min, tap water). After the antigen retrieval and permeabilization, non-specific binding sites were blocked with normal rabbit serum (Vector Laboratories, Burlingame, CA, USA). The slides were then incubated for 2 h with 1/50 diluted anti-neutrophil antibody, NIMP-R14 (Abcam, Cambridge, MA, USA). Antibody binding was detected using a biotinylated secondary antibody and ABC reagent from the Vectastain Elite ABC kit (Vector Laboratories). The slides were developed with peroxidase substrate solution, counterstained with hematoxylin, and mounted using VectaMount mounting medium (all from Vector Laboratories). The slides were observed and photographed with Moticam 3.0 MP Color Digital Camera (Motic).

### Immunofluorescence staining

Formalin-fixed colon tissues were embedded in paraffin and sectioned 4 to 5 μm. Slides were deparaffinized, rehydrated, and antigen retrieval was conducted on them as described above. After blocking non-specific binding sites with protein block serum free solution (Dako, Glostrup, Denmark), slides were incubated overnight at 4°C with 1/200 diluted FITC-conjugated F4/80 antibody (Biolegend, Inc., San Diego, CA, USA) and unconjugated CD3 antibody (Abcam, Cambridge, UK). In case of CD3, the slides were incubated for 1 h in dark room state with 1/2000 diluted FITC goat-anti rabbit 2^nd^ antibody (Bethyl Laboratories, Montgomery, TX, USA). Finally, the slides were mounted using Vectashield mounting medium with DAPI (Vector Laboratories, Burlingame, CA, USA), observed using Axioskop (Carl Zeiss, Oberkochen, Germany), and photographed with MetaMorph® software.

### Immunoblot analysis

Western blot analysis was performed as previously described [39]. The membranes were incubated overnight at 4°C with the primary antibodies including phospho-Thr^202^/Tyr^204^-ERK1/2 (#9101, 1:1000), ERK1/2 (#9102, 1:1000), phosphor-Ser^536^ NF-κB p65 (#3033, 1:1000) (Cell Signaling Technologies, Danvers, MA, USA), formyl peptide receptor-2 (FPR2) (sc-66901, 1:1000), and β-actin (sc-47778, 1:10000) (Santa Cruz Biotechnology Inc., Santa Cruz, CA, USA). After incubation, the membranes were washed 5 times for 10 ~ 15 min and then incubated with horseradish peroxidase-conjugated anti-rabbit (ADI-SAB-300-J) or anti-mouse (ADI-SAB-100-J) antibody (both from Enzo Life Sciences, Farmingdale, NY, USA) at room temperature for 1 ~ 2 h. Antigen-antibody complexes were visualized using enhanced chemiluminescence reagents (Thermo Scientific, Waltham, MA, USA) according to the manufacturer’s instructions.

### Enzyme-linked immunosorbent assay (ELISA)

Cytokine levels in colon tissue lysates were measured using mouse IL-6 ELISA Duo kits (R&D Systems, Minneapolis, MN, USA) according to the manufacturer's protocol. All assays were performed in triplicate, and data were expressed as mean ± SEM.

### Fluorescence-activated cell sorting (FACS) analysis

Total mouse blood cells were collected by capillary blood collection tubes with EDTA (VWR International, Radnor, PA, USA) and red blood cells (RBC) were selectively removed by 10-min incubation in RBC lysing buffer (Sigma-Aldrich). Then the blood cells were washed with PBS, fixed in 10% buffered neutral formalin solution (Sigma-Aldrich) at 37°C for 20 min, and incubated in blocking buffer (3% bovine serum albumin in PBS) at 37°C for 30 min. After blocking, the cells were incubated in the dark with the conjugated primary antibody, PE anti-mouse Ly-6G/Ly-6C (1:500) (Biolegend Inc.), for 1 h at room temperature. The cells were then washed twice, sorted and analyzed using an Accuri C6 flow cytometry system (Becton, Dickinson and Company (BD) bioscience, San Jose, CA, USA).

### Thin-layer chromatography (TLC)

To optimize a solid-phase extraction (SPE) method for fish oil fatty acids in serum, each 0.1 μg of fish oil fatty acids, including ARA, EPA, cis-7,10,13,16-docosatetraenoic acid (DTA), DHA, 12(*S*)-hydroxy-eicosatetraenoic acid (12HETE), 20-hydroxy-eicosatetraenoic acid (20HETE) and 5*S*,12*R*-dihydroxy-eicosatetraenoic acid-d4 (LB4-d4) (internal standard), were spiked into 0.5 mL fetal bovine serum (FBS) (WelGene, Daegu, Korea). The fish oil fatty acid standards were obtained from Sigma-Aldrich. Serum samples (0.5 mL) were acidified with 2 μL formic acid and mixed with 167 μL or 500 μL methanol (MeOH) to final concentrations of 25% or 50% MeOH, before loading onto 50 mg Sep-Pak® tC18 cartridges (Waters Co, Milford, MA, USA) activated with 100% MeOH and then equilibrated with the same concentration of MeOH in 0.4% formic acid. The effluent of each sample was re-loaded onto the tC18 cartridge three times, and the cartridges were washed with 10 mL of 25% or 50% MeOH in 0.4% formic acid. Residual buffer was removed by centrifugation at 1,000 × g for 2 min, and bound fatty acids were then eluted twice with 0.5 mL MeOH. The collected samples were dried *in vacuo* under N_2_ gas. The dried pellet was dissolved in 100 μL MeOH and spotted on a Silica gel 60 F254 (Merck KGaA, Darmstadt, Germany). The TLC silica gel was resolved by hexane-ethyl ether-formic acid (80:20:1, v/v/v) at ambient temperature. The TLC bands were visualized and photographed under UV light.

### Liquid chromatography–tandem mass spectrometry (LC-MS/MS)

Solid-phase extracts of fish oil fatty acid standards were dried under N_2_ gas and dissolved in 10 μL of 50% MeOH in 0.1% formic acid for tandem mass spectrometry analysis. Each 2 μL sample was manually analyzed using a Velos Pro Mass Instrument (Thermo Scientific) operated at a flow rate of 5 μL min^-1^ in a negative mode with the exit voltage of -4 kV. Full-scan survey was performed between *m/z* 150–400 and MS/MS spectra of the three most intense ions from the preview survey scan were acquired in the Ion Trap with the following options: isolation width, ± 0.8 *m/z*; collision energy, 35%; dynamic exclusion duration, 30 sec. To construct standard curves, extracted ion chromatograms were generated by Thermo Xcalibur Qual Browser version 2.1 with the precursor ion mass and mass tolerance of 100 ppm. To distinguish between 12HETE and 20HETE at *m/z* 319.25, their characteristic fragment ions at *m/z* 179 and 245 were detected by tandem mass spectrometry. A relative level of each fish oil fatty acid precursor ion was calculated by division with the level of internal standard LB4-d4 at *m/z* 339.49. LB4-d4 is an artificial material in which 4 hydrogen residues of natural leukotriene B_4_ were converted to deuterium (d) ions; it was used as an internal standard in the present study to achieve reliable quantification. To identify the unknown fatty acids in mouse sera, fish oil fatty acids were extracted from the samples collected by using capillary blood collection tubes, as described above. The N_2_ dried samples were dissolved in 20 μL of 50% methanol in 0.1% formic acid, and 10 μL portions were automatically analyzed on a Velos Pro Mass instrument equipped with an high-performance liquid chromatography and a Hypersil Gold C18 column, 1.9 μm particle size (Thermo Scientific). The chromatographic condition was a linear gradient from 25% to 100% MeOH in 0.1% formic acid for 30 min and then re-equilibrated with the initial buffer for 5 min. Mass data were acquisitioned by full mass survey scan, as described above, followed by 7 data-dependent scans of the most intense ions from the preview survey scan.

### Statistical analysis

Results were expressed as the mean ± SEMs. Group data were compared by 2-way analysis of variance followed by the multiple-comparison Bonferroni *t* test or 1-way analysis of variance followed by a Newman-Keuls post-hoc test to assess differences among groups. The nonparametric Mann-Whitney *U* test was used to compare histological difference. Otherwise, paired and 2-tailed Student’s *t*-test were used to compare results from the experiments. Statistical significance was defined by a *P* value of less than 0.05.

## Results

### Mucosal healing occurred after acute colitis

**Fig 1:**
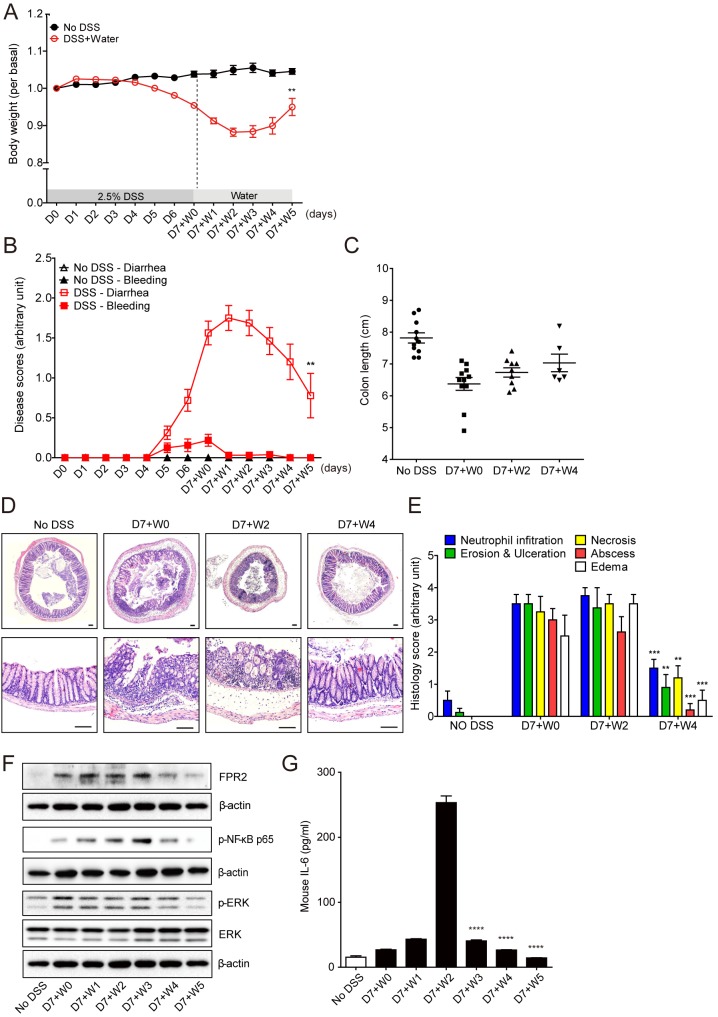
Intestinal inflammation ran a self-limited course and was gradually healed. Mice were fed 2.5% DSS for 7 days followed by tap water for 5 additional days. (A-C) Body weight changes (A), rectal bleeding and diarrhea (B), and colon length shortening (C) were monitored daily for a 12-day period. Rectal bleeding was scored by observation of blood in feces and the perianal area (0–5), whereas diarrhea was scored from normal feces to watery diarrhea (0–5). Results are mean ± SEM (n = 6~11 per group). **P<0.01. * indicates significance compared with D7+W2. (D, E) Representative images and histological scores of H&E stained colon from No DSS, D7+W0, D7+W2 and D7+W4 were presented. Original magnification: 20x in the upper panel and 100x in the lower panel. Scale bar, 100 μm. **P<0.01, ***P<0.001, and ****P<0.0001. * indicates significance compared with D7+W2. (F) Western blot analysis was performed using FPR2, p-NF-κB p65 and p-ERK antibodies. Total ERK and β-actin were used as a loading control. (G) Enzyme-linked immunosorbent assay was performed to measure mouse IL-6 levels. ****P<0.0001. * indicates significance compared with D7+W2. Results are mean ± SEM (n = 3 per group).

While a DSS-induced colitis model is a commonly used intestinal inflammation model, we hypothesized an intestinal wound healing model could be established by replacing DSS with water as previously described [36], and this model would recapitulate mucosal healing following acute colitis. To test this hypothesis, mice were provided 2.5% DSS in their drinking water for 7 days and tap water for 5 additional days, and then the symptoms of colitis including body weight loss, rectal bleeding, diarrhea, and colon length shortening were evaluated. About 5% of body weight loss when compared with day 0 (D0) was observed at day 7 (D7+W0) and weight loss continued for 2 ~ 3 additional days (the average weight loss was 12% at D7+W2). At D7+W2~D7+W3, body weight was gradually increased (Fig 1A). Rectal bleeding and diarrhea were also increased by DSS administration but steadily reduced upon switching to tap water (Fig 1B). Additionally, the length of the colon from the mice at D7+W0, D7+W2, and D7+W4 was measured. Colon length was decreased at D7+W0 as compared with control (No DSS group) and gradually increased afterwards even though there is no significant difference among groups (Fig 1C). Representative photographs of the colon at D7+W0, D7+W2, and D7+W4 were analyzed for histological changes. Distortion of crypts, submucosal edema, and immune cell infiltration were observed in the colon at D7+W0 and D7+W2 (Fig 1D). But the colon at D7+W4 showed intact histological structure which resembles that of healthy colon of no DSS-exposed mice (No DSS) (Fig 1D). Histological signs of inflammation including neutrophil infiltration, erosion & ulceration, necrosis, abscess, and edema were increased at D7+W0 and D7+W2, but the levels were significantly decreased at D7+W4 when compared with D7+W2 (Fig 1E). These results show that intestinal inflammation has been resolved and mucosal healing has occurred.

We next tested whether the levels of pro-inflammatory signaling pathways are altered during mucosal healing. FPR2 plays a role in host defense, immune regulation and inflammation [40, 41]. Besides, commensal bacteria modulate cellular signaling in intestinal epithelium by activating the ERK/MAPKs pathway in a FPR-dependent manner [42]. The protein expression of FPR2 was increased at D7+W0~D7+W3, but, as healing progressed, the expression of FPR2 was gradually decreased (Fig 1F). Transcription factor NF-κB controls pro-inflammatory gene expression and its activation is one of the most important steps in the development of intestinal inflammation [43]. NF-κB showed the highest activity at D7+W2~D7+W3 but the activity of NF-κB was gradually decreased afterwards (Fig 1F). Similarly, the phosphorylation of ERK was increased at D7+W0~D7+W3, but the activity was decreased afterwards (Fig 1F). Moreover, the expression level of pro-inflammatory cytokine, IL-6, was also increased at D7+W2 but the level was decreased afterwards (Fig 1G). These results indicate that mucosal healing is accompanied by reduction of NF-κB pathway, down-regulation of FPR2/ERK pathway, and inhibition of pro-inflammatory cytokine production.

### Endogenous mucosal healing program restored tissue homeostasis by regulating inflammatory cell infiltrations

**Fig 2:**
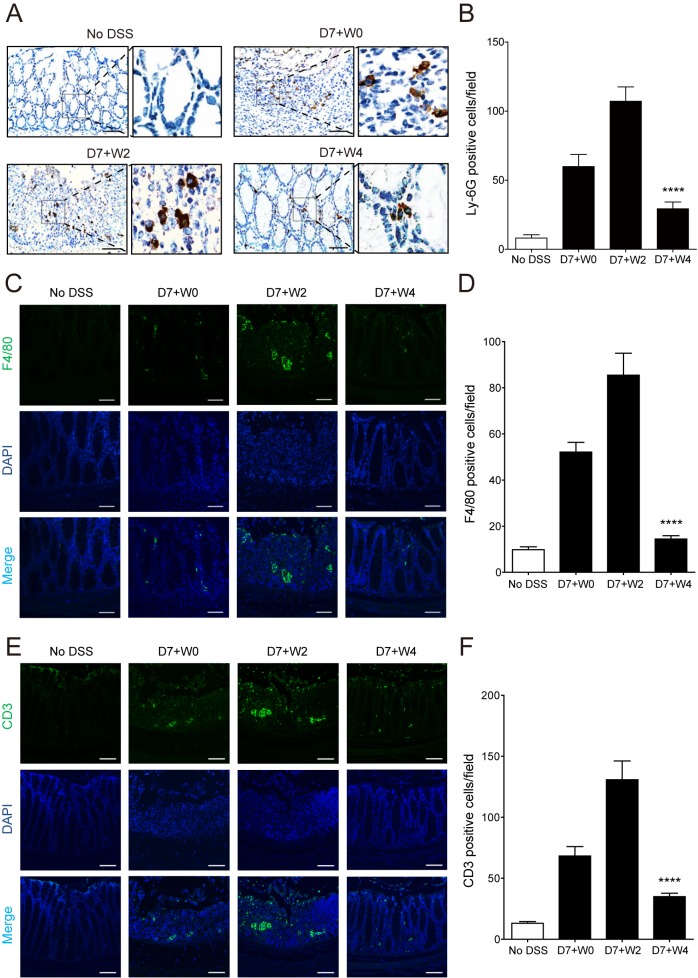
Reduction of inflammatory cell infiltration manifested the process of mucosal healing. Mice were fed 2.5% DSS for 7 days followed by tap water for 5 additional days and colonic tissues were prepared for immunohistochemical analysis. (A, C & E) Representative images of colon stained with Ly-6G (A), F4/80 (C), and CD3 (E) indicate the recruitment of neutrophils (brown), macrophages (green), and T cells (green), respectively. DAPI (blue) was used to indicate nucleus. Scale bar, 500 μm (A) and 100 μm (C & E). (B, D & F) For quantification, average of total cell number per field was counted in four independent photos of each group. ****P<0.0001. * indicates significance compared with D7+W2. Results are mean ± SEM (n = 4 per group).

Accumulation of neutrophils is associated with active inflammation states and is a common feature of human inflammatory diseases, suggesting a possible reduction of neutrophil infiltration during mucosal healing. In accordance with the hypothesis, recruitment of neutrophils, which was determined by the cell surface marker Ly-6G, significantly increased at D7+W0 and D7+W2 during inflammation (Fig 2A and 2B). At D7+W4, neutrophils left the colonic lamina propria and the number of neutrophils was substantially decreased in the colon (Fig 2A and 2B) suggesting termination of inflammation. Similarly, macrophages are also involved in resolution of inflammation by ingesting apoptotic cells, pathogens and debris. The number of infiltrated macrophages, which were stained by cell surface marker, F4/80, was increased at D7+W0 and D7+W2, but slightly decreased at D7+W4 by termination of inflammation (Fig 2C and 2D). Moreover, a recent study showed that suppression of CD3^+^ T cell activity by an oral CD3-specific antibody inhibited T cell-induced colitis in mice [44]. To determine the involvement of CD3^+^ T cells in mucosal healing, colon tissues were stained using FITC conjugated CD3 antibody. The number of CD3^+^ T cells was increased at D7+W0 and D7+W2, and was decreased at D7+W4 (Fig 2E and 2F). These results indicate that a wide array of inflammatory cells is infiltrated in the colonic lamina propria during colitis and may undergo apoptosis during the mucosal healing phase to restore tissue homeostasis.

### Endogenous lipid mediators were identified by lipidomics analysis

**Fig 3:**
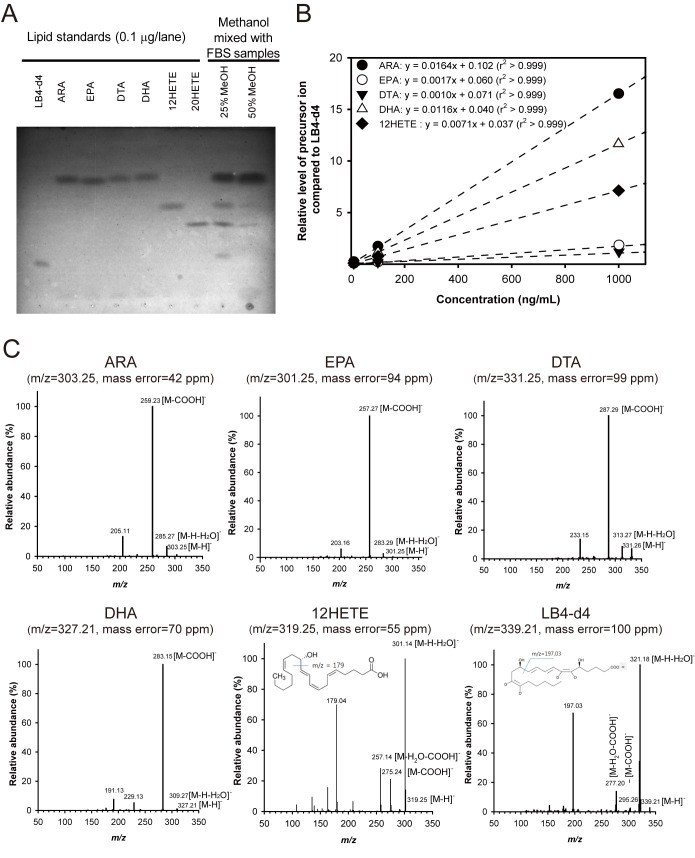
Standard curves for quantification of fish oil fatty acids were determined and tandem mass spectra of fish oil fatty acid standards were assigned. (A) To determine the efficiency of tC18-SPE of fish oil fatty acids spiked into fetal bovine serum, TLC was performed. (B) Standard curves for quantification of fish oil fatty acids relative to internal standard LB4-d4 by mass spectrometry was shown. (C) Results of tandem mass spectra of fish oil fatty acid standards were shown. Tandem mass spectra were assigned to the predicted fragment ions generated from collision-induced dissociation of the precursor ions. The major fragment ions of ARA: *m/z* 303.25 [M-H], *m/z* 285.27 [M-H-H_2_O], *m/z* 259.23 [M-COOH], and *m/z* 205.11. EPA: *m/z* 301.25 [M-H], *m/z* 283.29 [M-H-H_2_O], *m/z* 257.27 [M-COOH], and *m/z* 203.16. DTA: *m/z* 331.26 [M-H], *m/z* 313.27 [M-H-H_2_O], *m/z* 287.29 [M-COOH], and *m/z* 233.15. DHA: *m/z* 327.21 [M-H], *m/z* 309.27 [M-H-H_2_O], *m/z* 283.15 [M-COOH], *m/z* 229.13, and *m/z* 191.13. 12HETE: *m/z* 319.25 [M-H], *m/z* 301.14 [M-H-H_2_O], *m/z* 275.24 [M-COOH], *m/z* 257.14 [M-H_2_O-COOH], and *m/z* 179.04. LB4-d4: *m/z* 339.21 [M-H], *m/z* 321.18 [M-H-H_2_O], *m/z* 295.26 [M-COOH], *m/z* 277.20 [M-H_2_O-COOH], and *m/z* 197.03.

**Fig 4:**
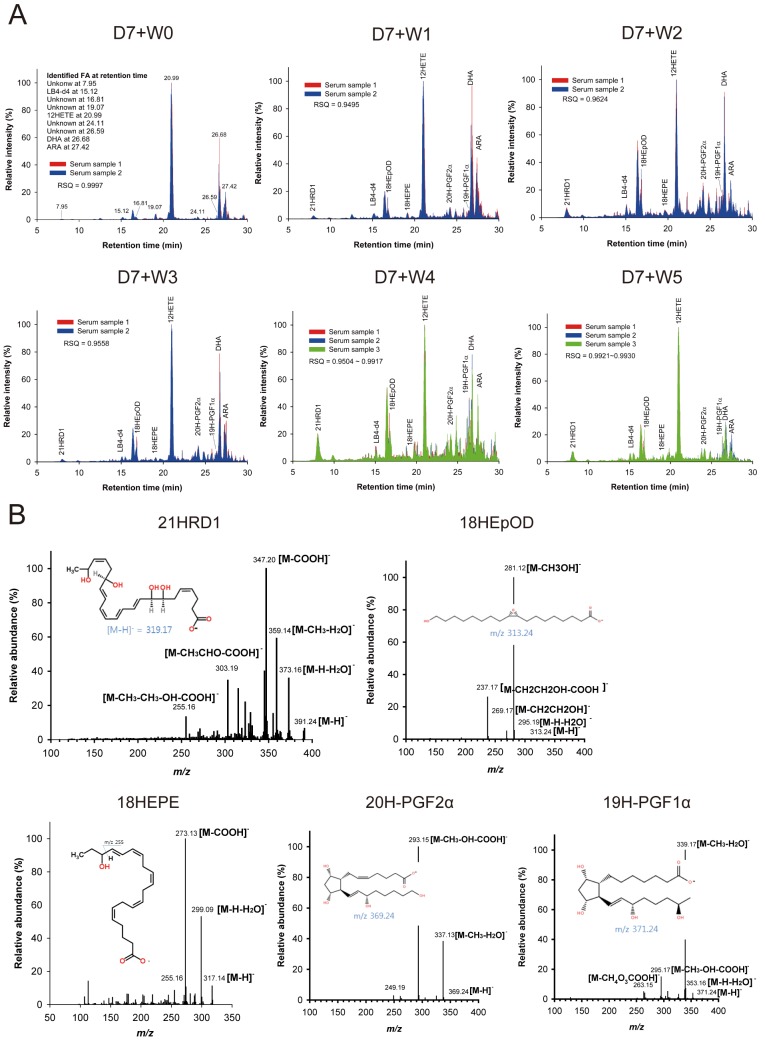
Quantitative analysis of fish oil fatty acids in mouse serum was performed by LC-MS/MS. (A) Base-peak chromatograms of lipid mediators in mouse sera were shown. LC-MS/MS analysis was conducted with two or three mouse sera obtained from D7+W0 to D7+W5 groups. Retention times of 21HRD1, LB4-d4, 18HEpOD, 18HEPE, 12HETE, 20H-PGF_2α_, 19H-PGF_1α_, DHA and ARA identified by tandem mass spectrometry are shown in the first chromatogram. The 9 lipid mediator peaks accounted for more than 80% in total ion chromatograms at all time-points. Base-peak patterns between chromatograms are analyzed by the *r*-squared value (RSQ): 0.9997 at D7+W0, 0.9495 at D7+W1, 0.9624 at D7+W2, 0.9558 at D7+W3, 0.9504~0.9917 at D7+W4, and 0.9921~0.9930 at D7+W5. (B) Unknown mass peaks in Fig 4A were assigned. Fish oil fatty acid components were confirmed by tandem mass spectra which were assigned to the fragment ions generated from collision-induced dissociation of the precursor ions. 21HRD1: *m/z* 391.24 [M-H], *m/z* 373.16 [M-H-H_2_O], *m/z* 359.14 [M-CH_3_OH_2_], *m/z* 347.20 [M-COOH], *m/z* 303.19 [M-CH_3_CHO-COOH], and *m/z* 255.16 [M-CH_3_-CH_3_-OH-COOH]. 18HEpOD: *m/z* 313.12 [M-H], *m/z* 295.19 [M-H_2_O], *m/z* 281.12 [M-CH_3_OH], *m/z* 269.17 [M-H-CH_2_CH_2_OH], and *m/z* 237.17 [M-H-CH_2_CH_2_OH-COOH]. 18HEPE: *m/z* 317.14 [M-H], *m/z* 299.09 [M-H-H_2_O], *m/z* 273.13 [M-COOH], and *m/z* 255.16. 20H-PGF_2α_: *m/z* 369.24 [M-H], *m/z* 337.13 [M-CH_3_-H_2_O], *m/z* 293.15 [M-CH_3_-OH-COOH], and *m/z* 249.19. 19H-PGF_1α_: *m/z* 371.24 [M-H], *m/z* 353.16 [M-H-H_2_O], *m/z* 339.17 [M-CH_3_-H_2_O], *m/z* 295.17 [M-CH_3_-OH-COOH], and *m/z* 263.15 [M-CH_4_O_3_COOH].

Our results in Figs 1 and 2 demonstrate that intestinal inflammation runs a definite limited course without exogenous treatment, suggesting a role of endogenous players in mucosal healing. Therefore, we hypothesized that endogenously produced lipids are involved in mucosal healing. To test this hypothesis, the lipid analysis by LC-MS/MS was conducted. Since the amount of lipids in the colon tissues is miniscule, serum from total blood was used for the analysis instead. First, lipid mediators within mouse serum were extracted by tC18-SPE. In order to optimize the SPE method, fish oil fatty acid standards spiked into sera were extracted with 25% or 50% MeOH solvent conditions. The total extraction efficiency confirmed by TLC was better with 25% MeOH than 50% MeOH solvent conditions (Fig 3A). Standard curves of fish oil fatty acids were determined relative to LB4-d4 (Fig 3B). The tandem mass spectra of fish oil fatty acid standards were assigned (Fig 3C). The endogenous levels of lipids in mouse serum obtained from the healing experiments were analyzed and quantitated by base-peak chromatograms (Fig 4A). Fish oil fatty acid lipid standards, such as ARA, DHA, 12HETE, LB4-d4, as well as several undescribed compounds were identified in LC-MS/MS analysis. Assignments of unknown mass peaks in mouse samples were conducted by tandem mass spectra in negative modes (Fig 4B). Tandem mass spectra of the unknown products under each peak at retention time (RT) = 7.95 min were matched to the predicted fragment ions from collision-induced dissociation of 21-hydroxy-resolvin D1 (21HRD1), the peak at RT = 16.81 min to 18-hydroxy-9,10-epoxyoctadecanoic acid (18HEpOD), the peak at RT = 19.07 min to 18-hydroxy-eicosapentaenoic acid (18HEPE), the peak at RT = 24.11 min to 9*S*,11*S*,15*S*,20-tetrahydroxy-5*Z*,13*E*-prostadienoic acid (20H-PGF_2α_), and the peak at RT = 26.59 min to 9*S*,11*R*,15*S*,19*R*-tetrahydroxy-13*E*-prostaenoic acid (19H-PGF_1α_) (Fig 4B). A total of 9 peaks of lipid mediators determined by LC-MS/MS were more than 80% in total ion chromatograms at all time-points during the progression of mucosal healing, but the relative abundance of each lipid mediator gradually changed.

### The levels of endogenous pro-inflammatory and pro-resolving lipid mediators were altered in the healing process

**Fig 5:**
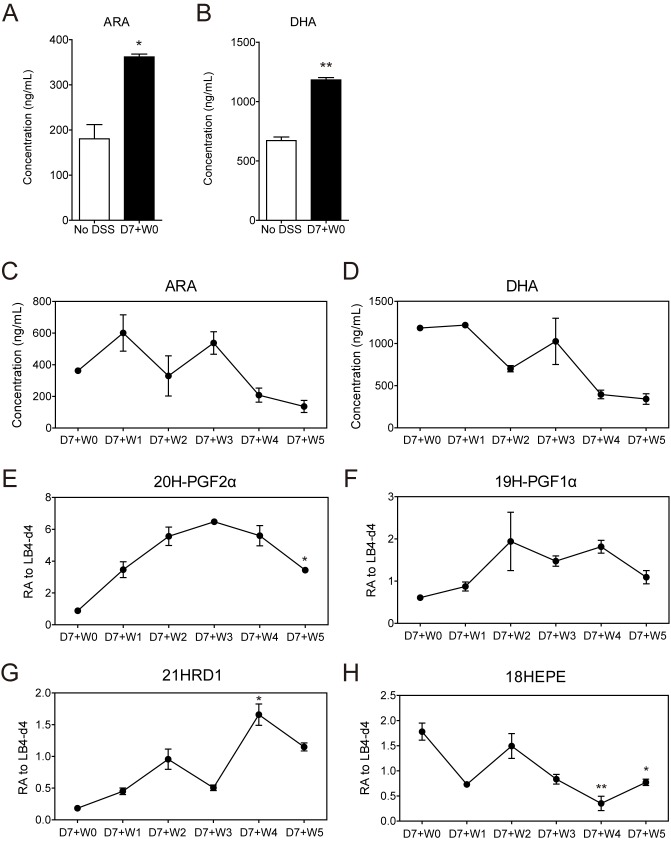
Pro-resolving lipid mediators were identified by LC-MS/MS. (A, B) The molar concentrations of ARA (A) and DHA (B) were determined by using standard curves. *P<0.05, and **P<0.01. * indicates significance compared with No DSS. (C-H) The concentrations of various lipids were changed during inflammation and healing. The concentrations ARA (C) and DHA (D) determined by standard values (curves). Relative abundance (RA) of 20H-PGF_2α_ (E), 19H-PGF_1α_ (F), 21HRD1 (G), and 18HEPE (H) was determined by division with the level of internal standard LB4-d4 spiked into sera. *P<0.05, and **P<0.01. * indicates significance compared with D7+W2.

Among various lipid mediators found in mouse serum obtained from the healing experiments, the levels of ARA and DHA, the major precursors of bioactive lipid mediators, were significantly increased in inflamed colon at D7+W0 when compared with ‘No DSS’ control (Fig 5A and 5B). This finding is consistent with the previous report that increased availability of the SPM precursors (n-3 PUFA) with feeding shortened the resolution interval in acute inflammation [45]. The elevated amounts of ARA and DHA were gradually decreased during the healing process and returned to the basal levels at D7+W5 (Fig 5C and 5D).

PGF_1α_ and PGF_2α_ are involved in initiation of inflammation. In lipid analysis, 20H-PGF_2α_, the metabolite of PGF_2α_ and 19H-PGF_1α_, the metabolite of prostacyclin, were detected (Fig 4A and 4B). The amount of ARA, the biosynthetic precursor of PGF_2α_, was decreased gradually, suggesting that ARA was used for synthesis of PGF_2α_ (Fig 5C). Consequently, the amount of 20H-PGF_2α_ was continuously increased until D7+W3 and then decreased afterwards (Fig 5E). Moreover, the amount of 19H-PGF_1α_ showed mild alterations with continued increase until D7+W2 and then decreased afterwards (Fig 5F). Therefore, the levels of inflammatory lipid mediators were reduced in the healing phase of colitis.

We further detected the changes in the relative levels of pro-resolving-associated lipid mediators. When the relative abundance of each component was calculated by division with the internal standard level of LB4-d4, the precursor of resolvin (Rv) D1, DHA was decreased and showed the lowest levels at D7+W4 suggesting indirectly an increase of RvD1 (Fig 5D). Consequently, an active metabolite of RvD1, 21HRD1, was continuously increased and was highly detected in D7+W4 (Fig 5G). Moreover, we also detected a gradual decrease in the precursor of RvEs, 18HEPE, during progression of resolution, although the active form of RvEs was not detected in LC-MS/MS (Fig 5H). Decreased levels of DHA and 18HEPE during the mucosal healing process may give a wide clue to an assumption that their well-known active products, RvD1 and RvEs, may play a role in this endogenous recovery process by inducing resolution.

### Administration of fish oil promoted mucosal healing in intestinal inflammation

**Fig 6:**
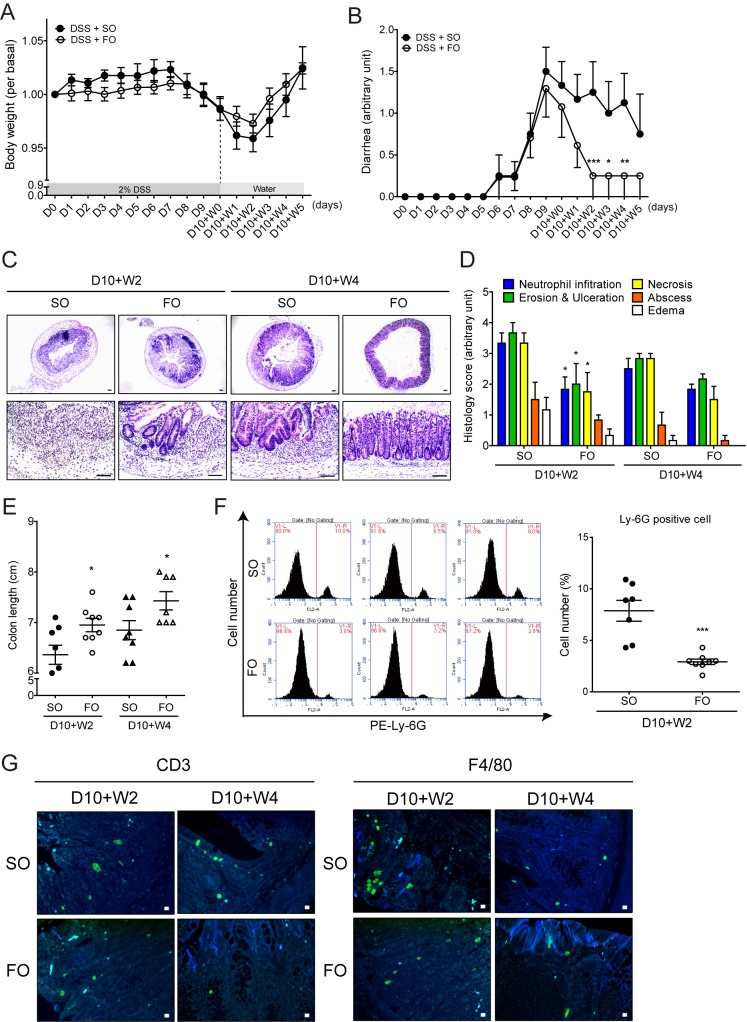
Mucosal healing was accelerated by fish oil supplementation. Mice were fed 2% DSS for 10 days followed by tap water for 5 additional days. During the last 5 days, the mice were supplemented daily with fish oil (FO) or sunflower oil (SO) via oral gavage. (A, B & E) Changes of body weight (A) and diarrhea (B) were monitored daily for a 15-day period. Results are mean ± SEM (n = 8 per group). *P<0.05, **P<0.01, and ***P<0.001. * indicates significance compared with SO group. (C, D) Representative images and histological score of H&E stained colon at D7+W2 and D7+W4 were presented. *P<0.05, * indicates significance compared with SO group at each time point. (E) Colon length was measured at D10+W2 and D10+W4. *P<0.05 * indicates significance compared with SO group at each time point. (F) FACS analysis was conducted to quantitatively determine the percentage of infiltrated neutrophils. PE anti-mouse Ly-6G/Ly-6C antibody was used for staining mouse neutrophils. Three representative histograms of SO and FO groups at D10+W2 were shown. ***P<0.001. * indicates significance compared with SO group. (G) Representative images of colon stained with CD3 and F4/80 antibodies indicate the recruitment of macrophages (green) and T cells (green), respectively. DAPI (blue) was used to indicate nucleus.

As our results in Figs 4 and 5 showed production of pro-resolving mediators during mucosal healing, we hypothesized that n-3 PUFA, a precursor of pro-resolving lipid mediators, can induce resolution in acute colitis. To test this hypothesis, fish oil supplementation experiments were conducted in a mucosal healing model. The mice were fed with 2% DSS for 10 days followed by tap water supplemented with 100 μl of fish oil or sunflower oil for 5 additional days. During the last 5 days, the mice were supplemented daily with fish oil or sunflower oil via oral gavage. Since fish oil contained 0.25 g/mL of DHA and EPA, the mice were fed 100 μg/g of DHA + EPA daily. DSS-induced inflammatory responses including body weight loss, diarrhea, colonic tissue damage and colon length shortening were alleviated upon switching to tap water in both fish oil and sunflower oil-fed groups (Fig 6A–6E). More importantly, mice in the fish oil group showed accelerated weight gain (Fig 6A), reduced diarrhea (Fig 6B), reduced tissue damage (Fig 6C), decreased histological scores (Fig 6D), and increased colon length (Fig 6E) when compared with the sunflower oil group. Likewise, neutrophil infiltration was significantly decreased in the fish oil group when compared with sunflower oil group when measured by FACS analysis using the neutrophil surface marker, PE-Ly-6G. Ly-6G positive cells (V1-R area in each data) of fish oil group were about 3% at D10+W2, whereas in sunflower oil group, they were about 8~10% (Fig 6F). Furthermore, infiltration of F4/80^+^ macrophages and CD3^+^ T cells in the inflamed sites were decreased by fish oil groups when compared with the sunflower oil group (Fig 6G). These results indicate that fish oil supplementation may help bring the healing time forward.

**Fig 7:**
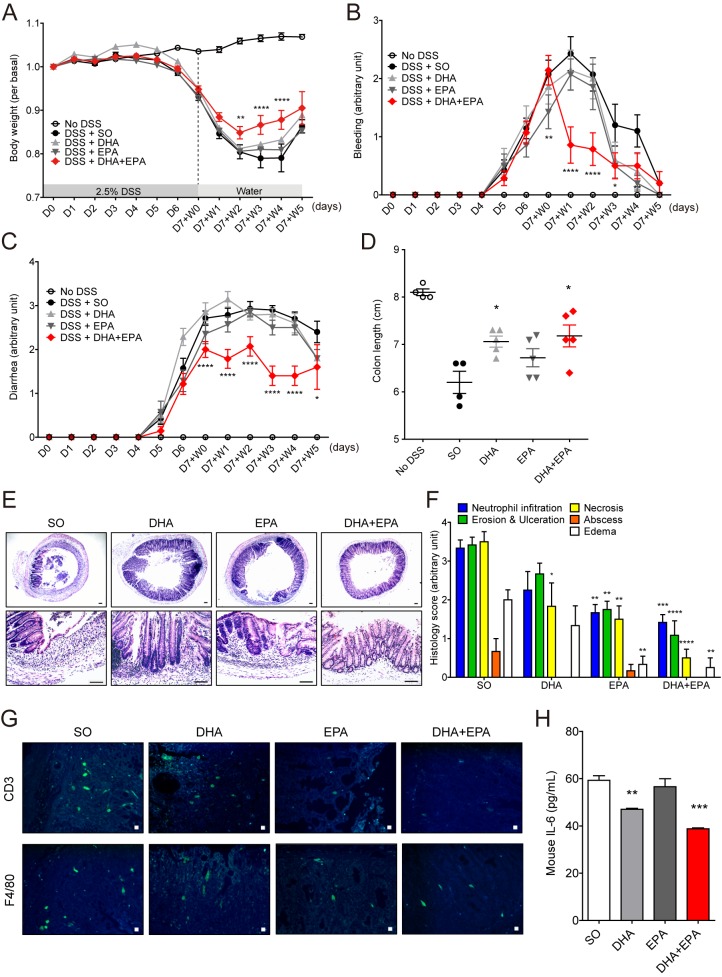
Mucosal healing was also promoted by DHA and EPA supplementation. Mice were fed 2.5% DSS for 7 days followed by tap water for 5 additional days. During the last 5 days, the mice were supplemented daily with DHA and/or EPA or sunflower oil (SO) via oral gavage. (A-C) Changes of body weight (A), rectal bleeding (B), and diarrhea (C) were monitored daily for a 12-day period. (D) Colon length shortening was measured at D7+W5. Results are mean ± SEM (n = 5~14 per group). *P<0.05, **P<0.01, ***P<0.001, and ****P<0.0001. * indicates significance compared with SO group. (E, F) Representative images and histological score of H&E stained colon at D7+W4 were shown. *P<0.05, **P<0.01, ***P<0.001, and ****P<0.0001. * indicates significance compared with SO. (G) Representative images of colon stained with CD3 and F4/80 at D7+W2 were presented. (H) Enzyme-linked immunosorbent assay was performed to measure mouse IL-6 levels. **P<0.01 and ***P<0.001. * indicates significance compared with SO. Results are mean ± SEM (n = 3 per group).

As exogenous supplementation of fish oil accelerated mucosal healing, we hypothesized that DHA and/or EPA supplementation would enhance the recovery process in colitis. To test this hypothesis, purified DHA and EPA supplementation experiments were conducted in a mucosal healing model. Mice were fed 2.5% DSS for 7 days followed by tap water for 5 additional days. During the last 5 days, the mice were supplemented daily with 50 μg/g DHA, 50 μg/g EPA, 25 μg/g DHA + 25 μg/g EPA or sunflower oil via oral gavage. DSS-induced inflammatory responses including body weight loss, bleeding and diarrhea, colon length shortening, and colonic tissue damage were alleviated upon switching to tap water in all the experimental groups (Fig 7A–7F). In agreement with the protective effects of fish oil supplementation (Fig 6), co-treatment of DHA and EPA accelerated the recovery process in each parameter analyzed. When compared with sunflower oil-fed mice, DHA and EPA-fed mice exhibited significantly more weight gain (Fig 7A) and significantly less bleeding and diarrhea (Fig 7B and 7C). In addition, the degree of reduction in colon length was significantly less in DHA alone or DHA and EPA-fed mice compared with the control (Fig 7D). Moreover, histological examinations of colonic tissues showed that EPA alone or DHA and EPA supplementation significantly reduced the histological severity score when compared with the sunflower oil supplementation (Fig 7E and 7F). Furthermore, during the recovery process the number of F4/80^+^ macrophages and CD3^+^ T cells in the inflamed sites was decreased by co-treatment of DHA and EPA when compared with the control treatment (Fig 7G). The expression levels of inflammatory cytokine, IL-6, were also reduced by DHA alone or DHA and EPA co-treatment when compared with the sunflower oil treatment (Fig 7H). These results suggest that exogenous DHA and EPA administration similar to fish oil supplementation could accelerate mucosal healing.

## Discussion

Finding an endogenous recovery mechanism of intestinal inflammation can be a new therapeutic strategy to help achieve stable remission in IBD. Predicting prognosis in patients with IBD is not easy as the clinical course of IBD is variable and the pattern of remission and relapse is unpredictable. For these reasons, complete remission is rarely achieved in patients with IBD and most patients need life-long uninterrupted treatment [46]. Therefore, elongation of the remission period without clinical symptoms is the ultimate goal to improve the quality of a patient’s life. Boosting endogenous remission pathways may be a more efficient treatment approach rather than inhibiting the systemic immune system.

The first principal object of this study was to characterize a self-limited recovery process. To mimic the remission phase of IBD, a wound healing model was first designed by modification of the DSS-induced colitis model [47]. The wound healing model was further used in several other studies to investigate a protective role or positive effects on regeneration of specific genes, cytokines and hormones. However, there is a lack of fundamental information on the self-limited healing process of intestinal inflammation without exogenous intervention. The present study characterized the endogenous mechanism for intestinal wound healing. Exposure to DSS induced the various clinical symptoms of colitis (Fig 1) including the recruitment of inflammatory cells (Fig 2). However, all mice were gradually recovered from the physiological alterations after termination of intestinal inflammation. Although an active inflammation response remained for 2~3 days after removing DSS, all clinical symptoms were improved gradually after D7+W3 and all mice achieved nearly complete tissue restoration at D7+W4 (Fig 1). This might be the outcome of a synergistic effect that combines the termination of the inflammation response and progression of the wound healing process.

Activation of the transcription factor NF-κB contributes to the development and maintenance of intestinal inflammation. In the western blot results, activation of NF-κB and ERK were significantly increased at the peak of inflammation but significantly decreased after the healing process progressed (Fig 1F). The expression of pro-inflammatory cytokine IL-6 was also increased in inflammatory conditions at D7+W2 but then rapidly decreased, returning to basal levels (Fig 1G). These results suggest that the endogenous recovery mechanism of the intestine may inhibit the pro-inflammatory response and also remove the stimuli for inflammation (i.e. luminal components crossing the barrier broken by the DSS).

Secondly, the present study shows alterations in the relative levels of endogenous lipid mediators in mouse sera at various healing phases after intestinal inflammation. PGF_2α_ is a major primary prostaglandin and increases vascular permeability to recruit immune cells to inflamed sites in acute inflammation [48]. The stable metabolite of prostacyclin, PGF_1α_, also has a role as potent vasodilator [49]. PGF_2α_ was gradually increased by induction of inflammation at D7+W2 and D7+W3 and then gradually decreased during the healing phase (Fig 5E). The number of infiltrated neutrophils also peaked at D7+W2 (Fig 2A and 2B), which was consistent with the relationship between prostaglandins production and neutrophil infiltration in a previous study of pleural inflammation using a mouse air pouch model [23]. Similarly, increased infiltration of neutrophils was gradually decreased during resolution after D7+W4 and termination of inflammation (Fig 2A and 2B).

Recent evidence suggests that alteration of lipid mediators is closely related to resolution of inflammation. During the initiation of inflammation, prostaglandins and leukotrienes increase, and then lipid mediator class switching occurs to terminate inflammation and to stimulate the resolution process [23]. Resolvins are the most well-known lipid mediators and have beneficial effects in several animal inflammation models such as peritonitis and asthma and colitis [19, 50, 51]. In intestinal inflammation, administration of RvE1 had beneficial effects by enhancing the survival rate and reducing pro-inflammatory gene expression in the 2,4,6-trinitrobenzenesulfonic acid-induced mouse colitis model [50]. Moreover, both RvD1 and RvD2 exhibited systemic anti-inflammatory effects in experimental colitis models by inhibiting NF-κB signaling [52]. These findings demonstrated the pharmacological activities of SPMs and provided rationale for use of SPMs for the treatment and prevention of many inflammatory states. But there is no evidence of endogenous alterations of pro-resolving mediators in intestinal inflammation.

In this study, without any exogenous administration of anti-inflammatory or pro-resolving mediators, a gradual increase in endogenously expressed 21HRD1, a tentative metabolite of RvD1, was observed in mouse serum during the healing phase; in contrast, its precursor DHA decreased as the healing process progressed (Fig 5D and 5G). Active metabolites of RvE1 were previously identified in both mouse and human [53, 54]. Among several metabolites, 19-hydroxy-RvE1 and 20-hydroxy-RvE1 were identified for their anti-inflammatory activities, and 20-hydroxy-RvE1 had leukocyte and neutrophils reduction activities as strong as those of RvE1 [54]. The previous studies suggested that the metabolites of resolvin may act as biomarkers of the resolution process and that 21HRD1 can be considered to be one of the active metabolites of RvD1. Therefore, the increased level of 21HRD1 in mouse serum indicates that resolution of intestinal inflammation is an actively regulated program. The inverse expression of prostaglandin also supports the hypothesis of a lipid mediator class switching mechanism in the resolution process.

Finally, because pro-resolving mediators were derived from n-3 PUFA, recent studies focused on the protective effect of n-3 PUFA during inflammation. One study showed that n-3 PUFA may contribute to a reduction of stem cell damage by modulating mediators of the colon stem cell niche [55]. In another study, nutritional supplementation of n-3 PUFA improved histological and clinical severity in the healing phase despite increased neutrophil infiltration [56]. However, there are no details on the physiological information of a positive effect of fish oil and n-3 PUFA in the healing process after acute colitis. In this study, we showed that fish oil supplementation during the resolution phase improved the clinical symptoms of colitis, and reduced the accumulation of neutrophils (Fig 6). Additionally, DHA and EPA treatment during the recovery process effectively alleviated the severity of colitis, reduced immune cell infiltration, and inhibited IL-6 production (Fig 7). These results suggest that fish oil and fish oil-derived n-3 PUFA can boost anti-inflammatory and pro-resolving activities in the intestine.

In conclusion, the resolution process following colitis involves alterations of endogenous levels of pro-resolving lipid mediators. Therefore, our findings extend our understanding of the existence of resolution process in intestinal inflammation and help us to develop new strategies to intervene during remission states of IBD patients.
